# Research on Material Basis and Quality Control of *Artemisiae argyi* Folium, a Traditional Medicinal and Edible Material

**DOI:** 10.1002/fsn3.70856

**Published:** 2025-09-01

**Authors:** Feifei Xie, Cong Ye, Haibao Qiu, Xiaoying Lu, Liyuan Qu, Zhizhou Ling, Zhenyu Li, Dongmei Sun, Minyou He, Lin Zhou, Wenhui Luo

**Affiliations:** ^1^ Guangzhou University of Traditional Chinese Medicine Guangzhou China; ^2^ Guangdong Yifang Pharmaceutical co., Ltd. Guangdong Provincial Key Laboratory of Traditional Chinese Medicine Formula Foshan China; ^3^ College of Food Science South China Agricultural University Guangzhou China; ^4^ Jiangxi Yifang Tianjiang Pharmaceutical co., Ltd. Nanchang China; ^5^ School of Chinese Materia Medica Guangdong Pharmaceutical University Guangzhou China

**Keywords:** *Artemisiae argyi* folium, chemometrics, component analysis, fingerprint, HPLC‐Q‐Exactive Orbitrap‐HRMS, quality control

## Abstract

*Artemisiae argyi* Folium (AAF) is a traditional medicinal and edible material. However, the pharmacologically active compounds and quality control of AAF have not been fully investigated. To solve these problems, we developed a novel method based on HPLC‐Q‐Exactive Orbitrap‐HRMS for rapid identification of chemical compounds in AAF. Forty‐five compounds were identified tentatively, mostly consisting of flavonoids and phenylpropanoids, and their fragmentation pathways were elucidated. Additionally, we present the first report on the HPLC‐UV fingerprints of AAF from genuine production areas, combined with chemometric analysis to elucidate the differences in the quality of AAF from different regions. Our results showed significant differences in the compounds of AAF from different production areas, and these variations could lead to inconsistencies in quality during large‐scale production. To ensure quality stability during the actual production process, it is essential to establish a method for rapid quantification of markers representing the potential differences in AAF quality. In this study, we developed a novel method called quantitative analysis of multicomponents by single marker for the simultaneous measurement of six phenylpropanoids and two flavonoids. Our study provides an analytical method and scientific basis for the standardized production, component analysis, and quality control of AAF as a functional food raw material.

## Graphical abstract

In this study, an innovative UHPLC‐Q‐Exactive Orbitrap‐HRMS technique was established for the rapid identification of flavonoids and phenylpropanoids as the main active components in *Artemisiae argyi* Folium. Furthermore, a single‐label quantitative method was developed to achieve the simultaneous detection of eight key components, thereby providing a scientific basis for its standardized production as a functional food ingredient.

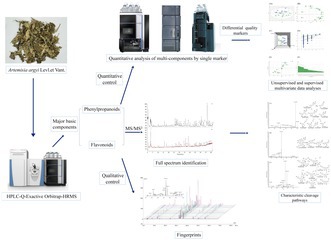

## 1 Introduction

*Artemisiae argyi* Folium (AAF), the dried leaves of the Asteraceae plant *Artemisia argyi* Levl. et Vant., is widely used as a traditional medicinal and edible material. *Artemisia argyi* is widely distributed in East Asia, including China, Mongolia, North Korea, South Korea, and Japan. In China, *A*. *argyi* is mainly found in Hubei, Hebei, Henan, and Anhui provinces (Abad et al. 2012). Varieties of *A*. *argyi* such as Wanai from Nanyang in Henan and Qiai from Qichun in Hubei are renowned for their excellent quality (Wang et al. 2024). In the food industry, AAF is used as a raw material for the traditional Chinese food Qing Dumpling and can also be used in tea, soup, and porridge to provide benefits such as enhanced disease resistance (Yu et al. 2023; Chen et al. 2024). Owing to its unique flavor, AAF is commonly used as a food additive in Japan and Mongolia (Xiao et al. 2019). In traditional medicine, moxa made from AAF has been used in moxibustion in China for thousands of years to treat dysmenorrhea, abdominal pain, and inflammation (Ahuja et al. 2018). Modern pharmacological studies have demonstrated the anticancer, anti‐inflammatory, and antioxidant activities of AAF, but most biological activities are only the results of in vitro experiments (Ye and Lai 2015; Wen et al. 2024). In recent years, research on AAF has majorly focused on the isolation and purification of chemical compounds and their pharmacological activities (Lee et al. 2018; Zhang et al. 2023). Studies have shown that some components including essential oils, flavonoids, organic acids, and coumarins have been identified in AAF (Song et al. 2019). Phenols, flavonoids, and organic acids in AAF contribute to its antioxidant, anti‐inflammatory, and antibacterial activities, respectively (Han et al. 2017). However, research on the chemical composition of AAF remains limited, with most studies focusing on volatile oils. Comprehensive analysis and characterization of the non‐volatile components of AAF have not yet been reported. Ultra‐high‐performance liquid chromatography coupled with Q‐Exactive Orbitrap high‐resolution mass spectrometry (HPLC‐Q‐Exactive Orbitrap‐HRMS) provides high sensitivity and resolution for qualitative analysis. This technique allows for the accurate and rapid acquisition of quasi‐molecular ions and their characteristic fragment ions. This method offers unique advantages for the analysis of the composition of complex systems and has been widely used in the functional component analysis of Chinese herbal medicines and functional foods (Dong et al. 2020). The present study aims to develop a method for the rapid identification of compounds in AAF via HPLC‐Q‐Exactive Orbitrap‐HRMS, thereby comprehensively elucidating the material basis of the pharmaceutical activities of AAF.

Under the influence of region, climate, and growing environment, the quality and composition of AAF can vary across different production areas. Presently, conducting a comprehensive evaluation of the active components of AAF poses a significant challenge, primarily due to the absence of fully developed uniform quality standards for AAF (Pan et al. 2025). Consequently, the quality of commercially available AAF remains inconsistent. Therefore, there is an urgent need to analyze the active components of AAF and establish suitable analytical methods for quality control. Fingerprint analysis allows for high‐resolution separation of complex components in medicinal and edible materials, to provide detailed and accurate chemical information and enable comprehensive characterization of the chemical compositions of the materials. This method can be used for authenticity verification, quality consistency evaluation, and product stability assessment (Liu et al. 2020; Su et al. 2022; Sun et al. 2019). However, fingerprint analysis is currently limited to qualitative assessment. The integration of fingerprint analysis with quantitative evaluation methods allows for a comprehensive and scientific evaluation of the quality of medicinal and edible raw materials (Luo et al. 2023). For conventional quantitative analysis, the use of the external standard method (ESM) for multicomponent quantification is limited by the unavailability and high cost of standard substances. In contrast, quantitative analysis of multicomponents by a single marker (QAMS) can allow for the simultaneous monitoring of multiple analytes using an inexpensive, stable, and readily available standard substance. This approach offers advantages in terms of cost‐effectiveness, efficiency, and speed (Yang et al. 2023). QAMS has been widely adopted for the simultaneous determination of multiple compounds in Chinese herbal medicines and has garnered extensive recognition in quality control research of Chinese medicinal materials (Wang et al. 2022). Therefore, we generated HPLC‐UV fingerprints of AAF from primary production areas in China and established a QAMS method for the simultaneous measurement of multiple characteristic compounds. These methods can be utilized for the quality control and evaluation of AAF as both a medicinal and edible material.

In this study, we developed a novel method based on HPLC‐Q‐Exactive Orbitrap‐HRMS for the rapid identification of chemical compounds in AAF. This method was employed to systematically uncover the material basis underlying the pharmacological activity of AAF. We investigated the characteristic fragmentation pathways of phenylpropanoids and flavonoids, and this laid the groundwork for identifying similar compounds in other medicinal and edible materials. Additionally, HPLC‐UV fingerprints were generated for AAF from two major production regions in China (Hubei and Henan provinces). These HPLC‐UV fingerprints, combined with chemometric analysis, allowed for the differentiation of AAF samples from these regions and provided insights into the causes of quality variation in actual production. We identified potential markers influencing quality variation and established a QAMS method for six phenylpropanoids and two flavonoids in AAF. This method greatly reduces analysis time, saves research costs, and enhances analytical efficiency. Our study offers a scientific and efficient analytical method and provides a solid foundation for the standardized production, component analysis, and quality control of AAF as a traditional medicinal and edible material.

## 2 Materials and Methods

### 2.1 Materials and Instruments

Eighteen batches of AAF samples were provided by enterprises from Henan and Hubei provinces, in China. The authenticity of these AAF samples was verified by Professor Sun Dongmei at Guangzhou University of Chinese Medicine. Chlorogenic acid, caffeic acid, schaftoside, rutin, apigenin, isoquercetin, cynaroside, luteoline, diosmetin, and casticin (all ≥ 92.2%) were purchased from the National Institutes for Food and Drug Control (Beijing, China). Neochlorogenic acid, cryptochlorogenic acid, vicenin 2, isochlorogenic acid B, naringenin, and artemetin (all ≥ 98%) were sourced from Nature Standard (Shanghai, China); neoschaftoside and centaureidin (both ≥ 95%) were obtained from Push Biotechnology (Chengdu, China). 1,5‐*O*‐dicaffeoylquinic acid (≥ 98.5%) was obtained from Must Biotechnology (Chengdu, China). Vicenin 3, isochlorogenic acid A, isochlorogenic acid C, and eupatilin (all ≥ 98%) were sourced from Weikeqi Biotech (Sichuan, China). Jaceosidin (≥ 98%) was purchased from Herbpurify (Chengdu, China). Irigenin (≥ 99.4%) was obtained from Lemeitian Medicine (Chengdu, China), and pectolinarigenin (≥ 98.85%) was obtained from Desite (Chengdu, China).

The instruments used in this study included the HPLC‐Q‐Exactive Orbitrap‐HRMS system and the Vanquish HPLC system (Thermo Fisher Scientific, USA), Waters H‐Class HPLC system (Waters, USA), Waters CORTECS C_18_ column (4.6 mm × 150 mm, 2.7 μm, Waters, USA), SHIMADZULC 40Dxs HPLC system (Shimadzu, Japan), ME204E analytical balance (0.1 mg readability, Mettler‐Toledo, Switzerland), XP26 balance (0.001 mg readability, Mettler‐Toledo, Switzerland), Milli‐Q water purification system (Merck, Germany), and KQ‐500DE numerical control ultrasonic cleaner (Kunshan Ultrasonic Instruments Co. Ltd., China). Methanol, acetonitrile, formic acid of chromatographic grade, and formic acid of mass spectrometry grade were purchased from Thermo Fisher Scientific (USA). All other reagents were of analytical grade.

### 2.2 Preparation of Sample Solutions

Exactly 0.2 g of the sample was added to 25 mL of 70% methanol and weighed. Ultrasonic extraction was conducted at 600 W power and 50 kHz frequency for 30 min. After the sample was cooled to room temperature, it was weighed again to determine weight loss, which was compensated for through the addition of the appropriate amount of the extraction solvent. The sample was then filtered through a 0.22 μm membrane, and the resulting filtrate was collected and analyzed by HPLC‐HRMS. This sample preparation method was also employed for QAMS analysis.

### 2.3 HPLC‐Q‐Exactive Orbitrap‐HRMS

The chromatographic analysis was conducted using a Waters CORTECS C_18_ column (4.6 mm × 150 mm, 2.7 μm), with acetonitrile (A) and 0.2% formic acid (B) as the mobile phases. The column oven temperature was maintained at 25°C, with a flow rate of 0.4 mL/min, UV detection wavelength set at 325 nm, and an injection volume of 2 μL. The elution gradient proceeded as follows: from 0 to 100 min, 8% to 100% (A); from 10 to 188 min, 10% to 144% (A); from 18 to 277 min, 14% to 200% (A); from 27 to 322 min, 20% to 211% (A); from 32 to 500 min, 21% to 400% (A); and from 50 to 622 min, 40% to 700% (A).

Mass spectrometry analysis was conducted via heated electrospray ionization, with a sheath gas flow rate of 35 arb, auxiliary gas flow rate of 10 arb, spray voltage set at 3.80 kV, S‐lens voltage at 50 V, and auxiliary temperature and capillary temperature both maintained at 350°C. The primary mass spectra were acquired in FT full scan mode with a resolution of 70,000. The secondary mass spectra were performed in data‐dependent scan mode with a resolution of 17,500. Full MS/dd‐MS^2^ discovery scan mode was employed in both positive and negative ion modes, with a scan range from *m*/*z* 120 to 1000 and optimal normalized collision energies of ±20 eV/±40 eV. For compound identification and analysis of fragmentation patterns of characteristic compounds, Xcalibur 2.1, Compound Discoverer 3.3, and the Thermo mzVault databases were utilized.

### 2.4 Generation of HPLC‐UV Fingerprints and Analysis of Quality Variation Across Production Areas

The chromatographic conditions were the same as those of HPLC‐Q‐Exactive Orbitrap‐HRMS. Isochlorogenic acid C, chosen for its moderate retention time, excellent peak shape, and high stability, served as the reference peak for normalizing common peaks. An 18 × 18 HPLC data matrix was established to record the peak areas of 18 compounds found across the 18 batches of AAF samples. Additionally, a chemometric approach based on principal component analysis (PCA) and orthogonal partial least squares discriminant analysis (OPLS‐DA) was applied to elucidate the factors contributing to variations in the quality of AAF from different production areas.

### 2.5 Development of a QAMS Method

The chromatographic conditions were consistent with those used in HPLC‐Q‐Exactive Orbitrap‐HRMS. Method validation included examining the effects of standard substance concentration, column lot number, column oven temperature, flow rate, and UPLC instrument on the relative correction factor (*f*) and relative retention time (RRT), with isochlorogenic acid C serving as the internal standard. The mean values of *f* and RRT were used for peak identification (Ling et al. 2022; Zhao et al. 2021). *f* was calculated using the formula: *f* = *f*_s_ */f*_i_ = (A_s_/C_s_)/(A_i_/C_i_), where A_s_ is the peak area of internal standard s, C_s_ is the concentration of internal standard s, A_i_ is the peak area of analyte i, and C_i_ is the concentration of analyte i. RRT was calculated as follows: RRT = *t*_i_/*t*_s_, where *t*_i_ denotes the retention time of analyte i, and *t*_s_ is the retention time of internal standard s. Finally, 18 batches of AAF from different origins were collected, and the content of each compound was determined via both QAMS and ESM methods. The accuracy of the QAMS method was verified by calculating the relative deviation (RD%) of the two methods.

## 3 Results and Discussion

### 3.1 Compound Identification and Fragmentation Patterns of Characteristic Compounds

#### 3.1.1 Compound Identification

**FIGURE 1:**
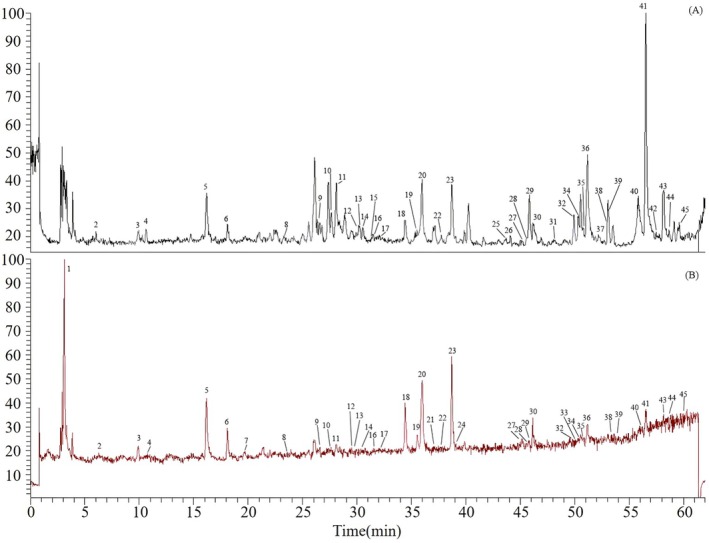
Total ion chromatogram of *Artemisiae argyi* Folium in the positive ion mode (A) and the negative ion mode (B). Numbers denote compounds.

HPLC‐Q‐Exactive Orbitrap‐HRMS was employed to generate total ion chromatograms of chemical compounds in AAF using both positive and negative ion modes (Figure 1). Accurate molecular masses obtained from both modes facilitated the determination of molecular formulas for various compounds in AAF. Moreover, the fragmentation patterns of the compounds were analyzed using MS^2^ spectra for preliminary structure identification. Subsequently, the tentatively identified compounds were confirmed using standard substances. The analysis revealed that AAF was rich in flavonoids and phenylpropanoids. Forty‐five compounds were identified tentatively (Table S1), and 26 compounds were confirmed through comparison with standard substances.

Phenylpropanoids displayed numerous characteristic fragment ions with relatively strong signals at a collision energy of ±20 eV. Caffeoylquinic acids containing multiple caffeoyl moieties exhibited a gradual loss of the caffeoyl groups, forming stable and distinct skeleton fragment ions. However, at collision energies higher than ±20 eV, the relative abundance of these skeleton fragment ions decreased, and in some cases, the ions were completely fragmented, hindering the structural identification of these caffeoylquinic acids. A collision energy of ±20 eV was insufficient for the complete retro Diels–Alder (RDA) reaction of the parent nuclei of flavonoids, resulting in few or no characteristic fragment ions. This limitation hindered effective structural identification. Therefore, in this study, a collision energy of ±40 eV was employed to elucidate the skeletons of flavonoid parent nuclei, while ±20 eV was utilized to determine the structures of substituents, thereby enhancing the accuracy of structural identification. Forty‐five compounds were identified tentatively, with 40 being phenylpropanoids or flavonoids. Phenylpropanoids were predominantly detected within the first 40 min of total ion chromatograms, while flavonoids were majorly observed in the subsequent 22 min. Consequently, we focused on a comprehensive analysis of the fragmentation patterns of these two groups of compounds.

#### 3.1.2 Identification of Phenylpropanoids and Their Fragmentation Patterns

Phenylpropanoids are natural compounds characterized by one or more C_6_–C_3_ units in their nuclei and are recognized for their pharmacological effects such as antibacterial, anti‐inflammatory, antiviral, and antihypertensive properties (Wang et al. 2024). The current study emphasizes the significance of phenylpropanoids in the pharmaceutical activity of AAF. Nine phenylpropanoids with relatively high abundance were identified tentatively, including neochlorogenic acid (Compound 3), chlorogenic acid (Compound 5), cryptochlorogenic acid (Compound 6), caffeic acid (Compound 7), isochlorogenic acid B (Compound 18), 1,5‐*O*‐dicaffeoylquinic acid (Compound 19), isochlorogenic acid A (Compound 20), isochlorogenic acid C (Compound 23), and 3,4,5‐tricaffeoylquinic acid (Compound 30). These phenylpropanoids, except for 3,4,5‐tricaffeoylquinic acid, were confirmed through comparison with standard substances. All compounds except for caffeic acid belonged to the caffeoylquinic acids family. The cleavage of ester bonds in caffeoylquinic acids in the negative ion mode produced abundant characteristic fragment ions such as [M–caffeoyl–H]^−^ and [caffeic acid–H]^−^. Therefore, the MS^2^ spectra in the negative ion mode were utilized for the analysis of fragmentation patterns.

**FIGURE 2:**
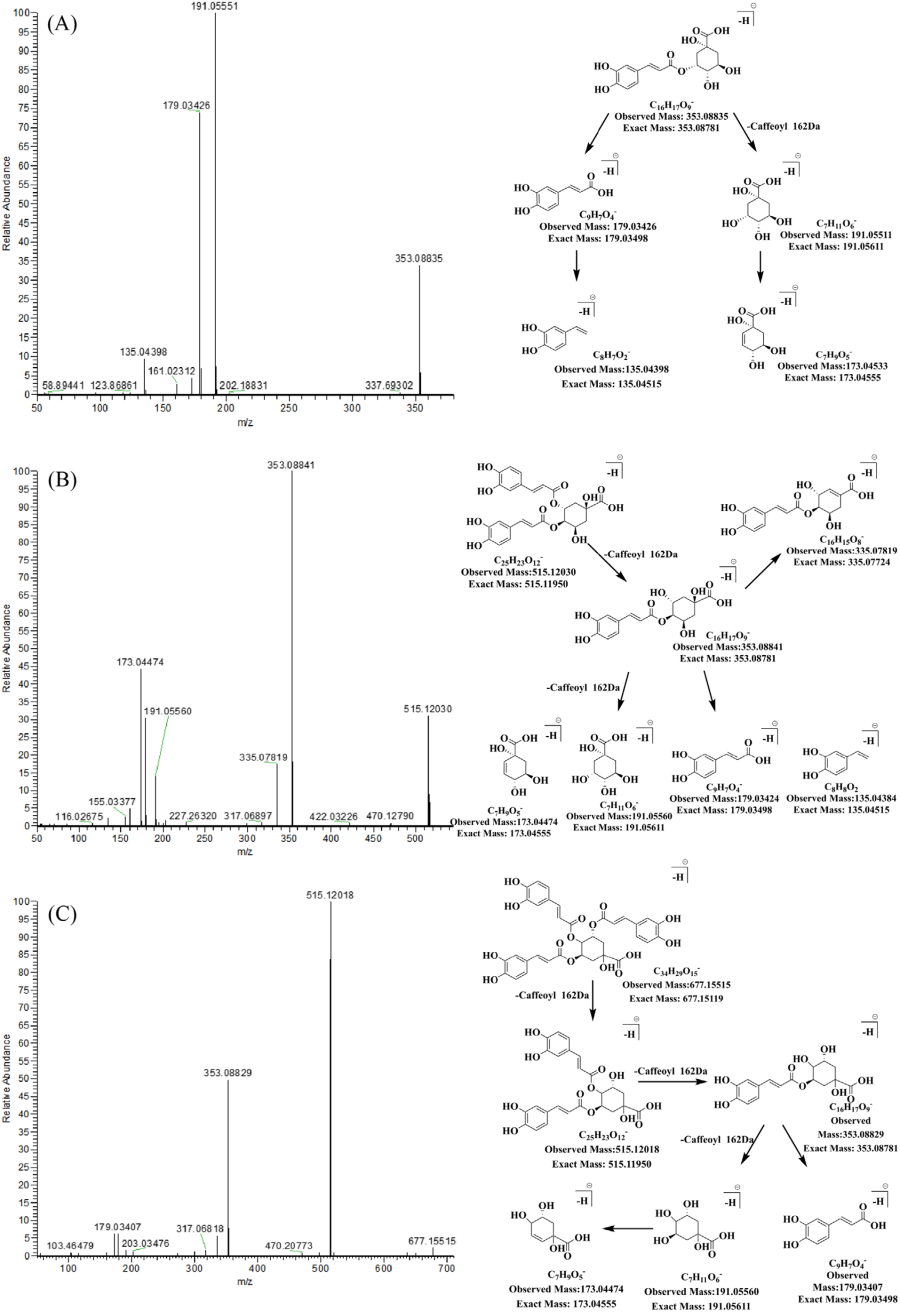
MS^2^ spectra and fragmentation patterns of selected phenylpropanoids in *Artemisiae argyi* folium in the negative ion mode. (A–C) Stand for neochlorogenic acid, isochlorogenic acid B, and 3,4,5‐tricaffeoylquinic acid, respectively.

Neochlorogenic acid, chlorogenic acid, and cryptochlorogenic acid are monocaffeoylquinic acids formed through the esterification of one molecule of caffeic acid and one molecule of quinic acid. These compounds are structural isomers distinguished by the position of esterification. For example, neochlorogenic acid (Figure 2A) exhibited quasi‐molecular ion peaks at *m*/*z* 355.10239 [M–H]^−^ and *m*/*z* 353.08835 [M–H]^−^, along with MS^2^ fragment ions at *m*/*z* 191.05551, 179.03426, 173.04533, and 135.04398 in the negative ion mode. The proposed fragmentation pathway involved [M–H]^−^ losing a caffeoyl moiety, resulting in the quinic acid skeleton fragment ion [M–caffeoyl–H]^−^ at *m*/*z* 191.05551 (base peak), followed by the loss of a water molecule to yield *m*/*z* 173.04445. The fragment ion of the caffeoyl moiety appeared as [caffeic acid–H]^−^ at *m*/*z* 179.03426, which further lost a CO_2_ molecule to yield *m*/*z* 135.04398. Chlorogenic acid and cryptochlorogenic acid exhibited similar fragmentation pathways, but the relative abundance of characteristic fragment ions varied owing to differences in esterification positions. The base peaks for neochlorogenic acid and chlorogenic acid were observed at *m*/*z* 191.05551 and 191.05556, respectively, while the base peak for cryptochlorogenic acid was at *m*/*z* 173.04474. The relative abundance of *m*/*z* 179 was significantly lower for chlorogenic acid (1.09%) than for neochlorogenic acid (52.54%). Therefore, neochlorogenic acid, chlorogenic acid, and cryptochlorogenic acid can be differentiated through a comparison of the relative abundance of their base peaks and the characteristic fragment ion *m*/*z* 179.

Isochlorogenic acid B, 1,5‐*O*‐dicaffeoylquinic acid, isochlorogenic acid A, and isochlorogenic acid C are dicaffeoylquinic acids formed through the esterification of two molecules of caffeic acid and one molecule of quinic acid. For example, the quasi‐molecular ion peaks of isochlorogenic acid B were [M + H]^+^ at *m*/*z* 517.13452 and [M–H]^−^ at *m*/*z* 515.12030 (Figure 2B). In the negative ion mode, the MS^2^ fragment ions of isochlorogenic acid B included *m*/*z* 353.08841 (base peak), 335.07819, 191.05560, 179.03424, 173.04474, and 135.04384. The proposed fragmentation pathway involves [M–H]^−^ sequentially losing two caffeoyl moieties, resulting in characteristic fragment ions [M–caffeoyl–H]^−^ at *m*/*z* 353.08841 and [M–2caffeoyl–H]^−^ at *m*/*z* 191.05560. Additionally, [M–H]^−^ loses one caffeoyl moiety and subsequently a water molecule to produce the characteristic fragment ion [M–caffeoyl–H_2_O–H]^−^ at *m*/*z* 335.07819. The fragmentation pathways of other fragment ions were analogous to those observed for monocaffeoylquinic acids. The base peak of isochlorogenic acid B, isochlorogenic acid A, and isochlorogenic acid C was observed at *m*/*z* 353, with the relative abundance of the characteristic fragment ion at *m*/*z* 173 following the order: isochlorogenic acid B (44.28%) > isochlorogenic acid C (29.35%) > isochlorogenic acid A (1.26%). In contrast, the base peak of 1,5‐*O*‐dicaffeoylquinic acid appeared at *m*/*z* 191.05557. Therefore, through a comparison of the base peaks and the relative abundance of the characteristic fragment ion *m*/*z* 173 among dicaffeoylquinic acids, distinctions can be drawn between isochlorogenic acid B, 1,5‐*O*‐dicaffeoylquinic acid, isochlorogenic acid A, and isochlorogenic acid C. These fragmentation patterns were validated using standard substances. The 3,4,5‐tricaffeoylquinic acid (Figure 2C) is a tricaffeoylquinic acid formed through the esterification of three molecules of caffeic acid with one molecule of quinic acid. In the negative ion mode, the quasi‐molecular ions of 3,4,5‐tricaffeoylquinic acid were [M + H]^+^ at *m*/*z* 679.16644 and [M–H]^−^ 677.15515. The proposed fragmentation pathway involves [M–H]^−^ sequentially losing three caffeoyl moieties to generate characteristic fragment ions: [M–caffeoyl–H]^−^ at *m*/*z* 515.12018, [M–2caffeoyl–H]^−^ at *m*/*z* 353.08829, and [M–3caffeoyl–H]^−^ at *m*/*z* 191.05644. The fragmentation pathways of other characteristic fragment ions were identical to those observed for dicaffeoylquinic acids. These fragmentation patterns are generally applicable for the structural analysis of phenylpropanoids in medicinal and edible materials via mass spectrometry in the negative ion mode.

#### 3.1.3 Identification of Flavonoids and Their Fragmentation Patterns

**FIGURE 3:**
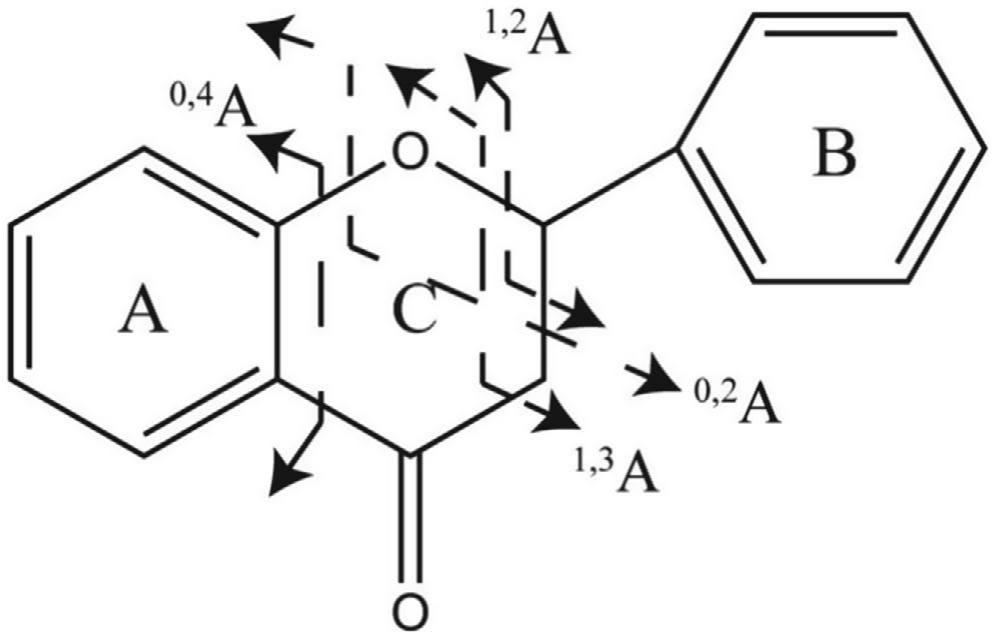
Nomenclature of fragment ions of flavonoid parent nucleus and C ring from RDA reaction.

Flavonoids are compounds with 2‐phenylchromanone as the parent nucleus and broadly refer to compounds with two benzene rings (A ring and B ring) linked by three carbon atoms (C ring) (Figure 3). The current study identified flavonoids as the predominant active compounds of AAF. These compounds exhibit diverse pharmacological activities, such as antioxidant, anti‐inflammatory, and antitumor properties (Wang et al. 2024). Specifically, 31 flavonoids were identified tentatively, including flavones, flavonols, flavonoid *O*‐glycosides, and flavonoid *C*‐glycosides. Of these compounds, vicenin 2 (Compound 8), schaftoside (Compound 9), neoschaftoside (Compound 10), vicenin 3 (Compound 11), rutin (Compound 12), isoquercetin (Compound 16), cynaroside (Compound 17), luteolin (Compound 28), apigenin (Compound 32), naringenin (Compound 33), diosmetin (Compound 35), irigenin (Compound 37), centaureidin (Compound 39), artemetin (Compound 42), chrysosplenetin B (Compound 43), and pectolinarigenin (Compound 45) were previously confirmed using standard substances. Neoschaftoside, vicenin 3, irigenin, and pectolinarigenin were newly identified tentatively in AAF during this study.

**FIGURE 4:**
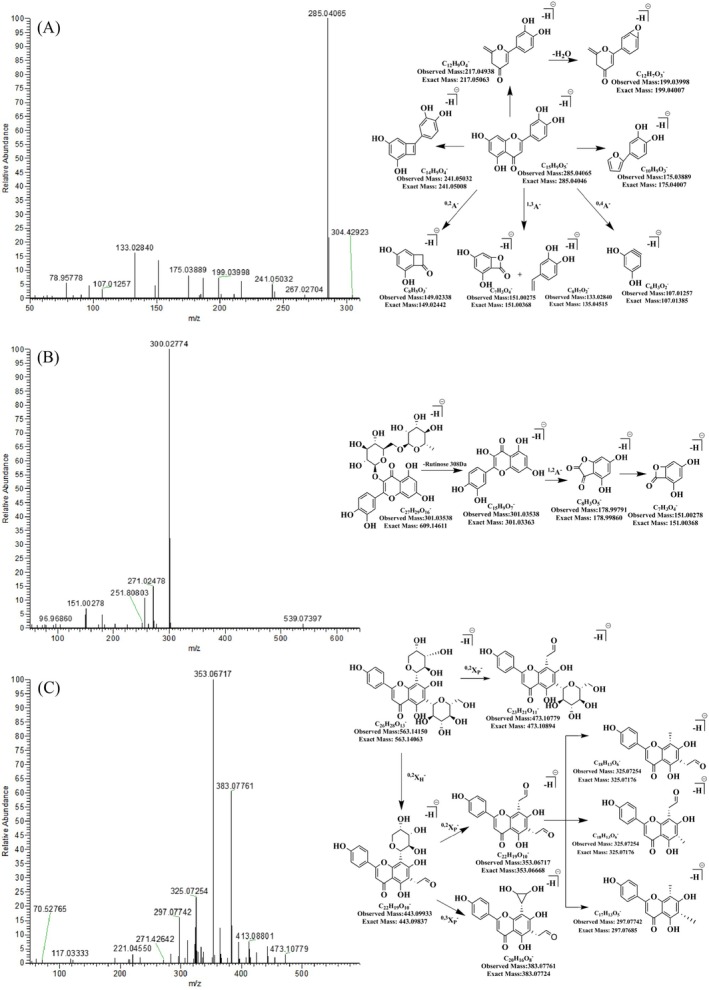
MS/MS^2^ spectra and fragmentation patterns of selected flavonoids in *Artemisiae argyi* Folium in the negative ion mode. (A–C) Represent luteolin, rutin, and schaftoside, respectively; Xp and X_H_ denote the ring opening of pentose and hexose, and A denotes the RDA reaction.

The RDA reaction is the characteristic fragmentation pathway of flavonoids. In AAF, flavonoids undergo cleavage at the 1,3, 0,2, and 0,4 bonds of the C ring in the negative ion mode, resulting in the formation of ^1,3^A, ^0,2^A, and ^0,4^A RDA fragment ions, which are typically abundant. The quasi‐molecular ion peaks of luteolin (Figure 4A) and apigenin were [M–H]^−^ at *m*/*z* 285.04065 and [M–H]^−^ at *m*/*z* 269.04572, respectively. Because they had the same A ring and C ring structures, luteolin and apigenin produced the same RDA fragment ions. In the negative ion mode, ^1,3^A^−^ fragment ions were observed at *m*/*z* 151.00275 and 151.00244 for luteolin and apigenin, respectively. The ^0,2^A^−^ fragment ions appeared at *m*/*z* 149.02338 and 149.02330, while the ^0,4^A^−^ fragment ions were detected at *m*/*z* 107.01257 and 107.01252 for luteolin and apigenin, respectively. In the positive ion mode, luteolin and apigenin only produced ^1,3^A^+^ fragment ions at *m*/*z* 153.01894 and 153.01840, respectively. Therefore, analyzing flavonoids in the negative ion mode is recommended to enhance the detection of abundant fragment ions resulting from the RDA reaction.

The study demonstrated that flavonoid *O*‐glycosides in AAF feature flavone or flavonol skeletons associated with one or more sugar moieties via C–O bonds. Under mass spectrometry conditions, flavonoid *O*‐glycosides progressively lose the entire glycosyl fragment, leaving behind the flavonoid aglycone, which undergoes the RDA reaction in the C ring. For example, rutin (Figure 4B), which was confirmed using a standard substance, exhibited quasi‐molecular ion peaks of [M + H]^+^ at *m*/*z* 611.16125 and [M–H]^−^ at *m*/*z* 609.14697. In the negative ion mode, MS^2^ fragment ions were observed at *m*/*z* 301.03363, 300.02774, 178.99791, and 151.00278. The proposed fragmentation pathway involves [M–H]^−^ losing a rutinose molecule, resulting in the flavonol nucleus fragment ion at *m*/*z* 301.03363. Subsequently, the flavonoid aglycone undergoes an RDA reaction, generating the ^1,2^A^−^ fragment ion at *m*/*z* 178.99791, followed by the loss of a CO molecule to yield *m*/*z* 151.00278. In the positive ion mode, only the fragment ion of the flavonol nucleus at *m*/*z* 303.05005 was observed. A similar fragmentation pattern was observed for isoquercetin. The quasi‐molecular ion peaks of isoquercetin were [M + H]^+^ at *m*/*z* 465.10333 and [M–H]^−^ at *m*/*z* 463.08887. In the negative ion mode, isoquercetin generated the flavonol nucleus fragment ion at *m*/*z* 301.03412 and the ^1,2^A^−^ fragment ion at *m*/*z* 178.99759. Subsequently, it lost one CO molecule to produce *m*/*z* 151.00258. In the positive ion mode, only the flavonol nucleus fragment ion at *m*/*z* 303.05002 was observed. Fragment ions resulting from deglycosylation and the RDA reaction were observed in the negative ion mode for flavonoid *O*‐glycosides in AAF, whereas fragment ions from the RDA reaction were absent in the positive ion mode. Therefore, it is recommended to analyze flavonoid *O*‐glycosides in AAF using the negative ion mode for optimal detection of these characteristic fragmentation patterns.

Flavonoid *C*‐glycosides in AAF feature flavonoid skeletons with five‐ or six‐carbon sugars directly related to the flavonoid nucleus via C–C bonds. Different from flavonoid *O*‐glycosides, which lose entire sugar moieties, negative‐ion‐mode fragmentation of flavonoid *C*‐glycosides in AAF revealed fragment ions [M–H–60]^−^, [M–H–90]^−^, and [M–H–120]^−^. These ions originated from a ring‐opening reaction of the sugar moiety attached to the C‐6 or C‐8 position of the A ring in the flavonoid nucleus. Specifically, [M–H–120]^−^ originated from a fragment ion resulting from the ring‐opening of a hexose rather than a pentose. The aglycone of flavonoid *C*‐glycosides in AAF did not undergo the RDA reaction. For example, schaftoside, confirmed using a standard substance, exhibited quasi‐molecular ions [M + H]^+^ at *m*/*z* 565.15590 and [M–H]^−^ at *m*/*z* 563.14150 (Figure 4C). In the negative ion mode, MS^2^ fragment ions of schaftoside were observed at *m*/*z* 473.10779, 443.09933, 383.07761, 353.0671, 332.80417, 325.07254, and 297.07742. The proposed fragmentation pathway involves the 0,2 cleavage of the hexose at C‐8 of [M–H]^−^ to produce the fragment ion [M–H–90]^−^ at *m*/*z* 473.10779. Additionally, the hexose at C‐6 of [M–H]^−^ may undergo 0,2 cleavage to produce the fragment ion [M–H–120]^−^ at *m*/*z* 443.09933. Moreover, [M–H]^−^ undergoes 0,2 cleavage of the hexose and 0,3 cleavage of the pentose to produce [M–H–120–60]^−^ at *m*/*z* 383.07761 and 0,2 cleavage of the hexose and 0,2 cleavage of the pentose to produce [M–H–120–90]^−^ at *m*/*z* 353.06717. These are followed by the loss of a CO molecule to yield *m*/*z* 325.07254 or the loss of two CO molecules to yield *m*/*z* 297.07742. However, fragment ions resulting from the ring‐opening of sugars in flavonoid *C*‐glycosides were absent in the positive ion mode. Therefore, it is recommended that the identification of flavonoid *C*‐glycosides in AAF be conducted in the negative ion mode. These fragmentation patterns can be applied for the structural analysis of flavonoids in medicinal and edible materials via mass spectrometry in the negative ion mode.

### 3.2 Generation of HPLC‐UV Fingerprints and Quality Control of AAF Samples From Genuine Production Areas

#### 3.2.1 Generation of HPLC‐UV Fingerprints

**FIGURE 5:**
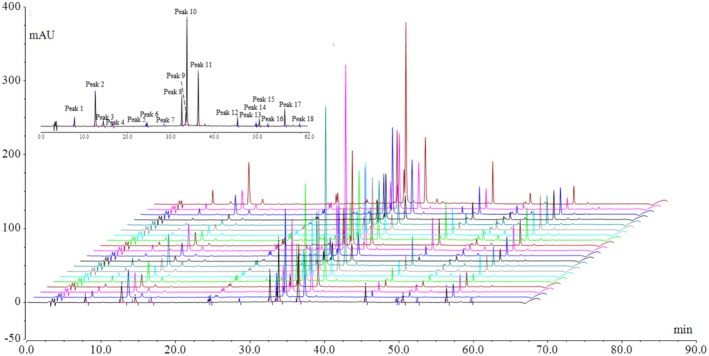
HPLC‐UV fingerprints of *Artemisiae argyi* Folium from primary production areas in China. Peaks 1–18 are shared among sample batches. Peaks 1–11 and 13–18 were confirmed using reference standards as neochlorogenic acid, chlorogenic acid, cryptochlorogenic acid, caffeic acid, schaftoside, neoshaftoside, rutin, isochlorogenic acid B, 1,5‐O‐dicaffeoylquinic acid, isochlorogenic acid A, isochlorogenic acid C, apigenin, diosmetin, jaceosidin, centaureidin, eupatilin, and pectolinarigenin, respectively. Peak 12 was identified using high‐resolution mass spectrometry as 3,4,5‐tricaffeoylquinic acid.

According to HPLC‐Q‐Exactive Orbitrap‐HRMS analysis, phenylpropanoids and flavonoids were the predominant compounds in AAF. Furthermore, HPLC‐UV fingerprints were generated for 18 batches of AAF samples from Henan and Hubei via HPLC‐DAD. The HPLC‐UV fingerprints were used for the quality control and assessment of AAF from these production areas (Figure 5). The HPLC‐UV fingerprints exhibited resolutions greater than 1.5 for major peaks, symmetric peak shapes (asymmetry factor, 0.98–1.15), high response values, and generally high separation efficiency. This method is comprehensive, stable, and highly specific. The method is thus capable of assessing the overall quality of AAF from different production areas. This method represents a new scientific approach for the quality control of AAF. Eighteen shared characteristic peaks were identified using the Chinese Medicine Chromatographic Fingerprint Similarity Evaluation System (2012 Edition). All of the peaks were confirmed to correspond to flavonoids or phenylpropanoids. This is the first report on the HPLC‐UV fingerprints of AAF from major production areas in China, and good separation was achieved.

#### 3.2.2 Chemometric Analysis of Potential Quality Markers for AAF From Different Production Areas

Owing to variations in soil environment, climate, and altitude, significant differences exist in the composition of AAF from different regions. Currently, the quality control standards for AAF production are inadequate. To ensure stable AAF quality, it is crucial to identify the factors contributing to these regional quality differences and to screen for markers indicative of these variations. This approach will enhance the evaluation and control of AAF quality.

**FIGURE 6:**
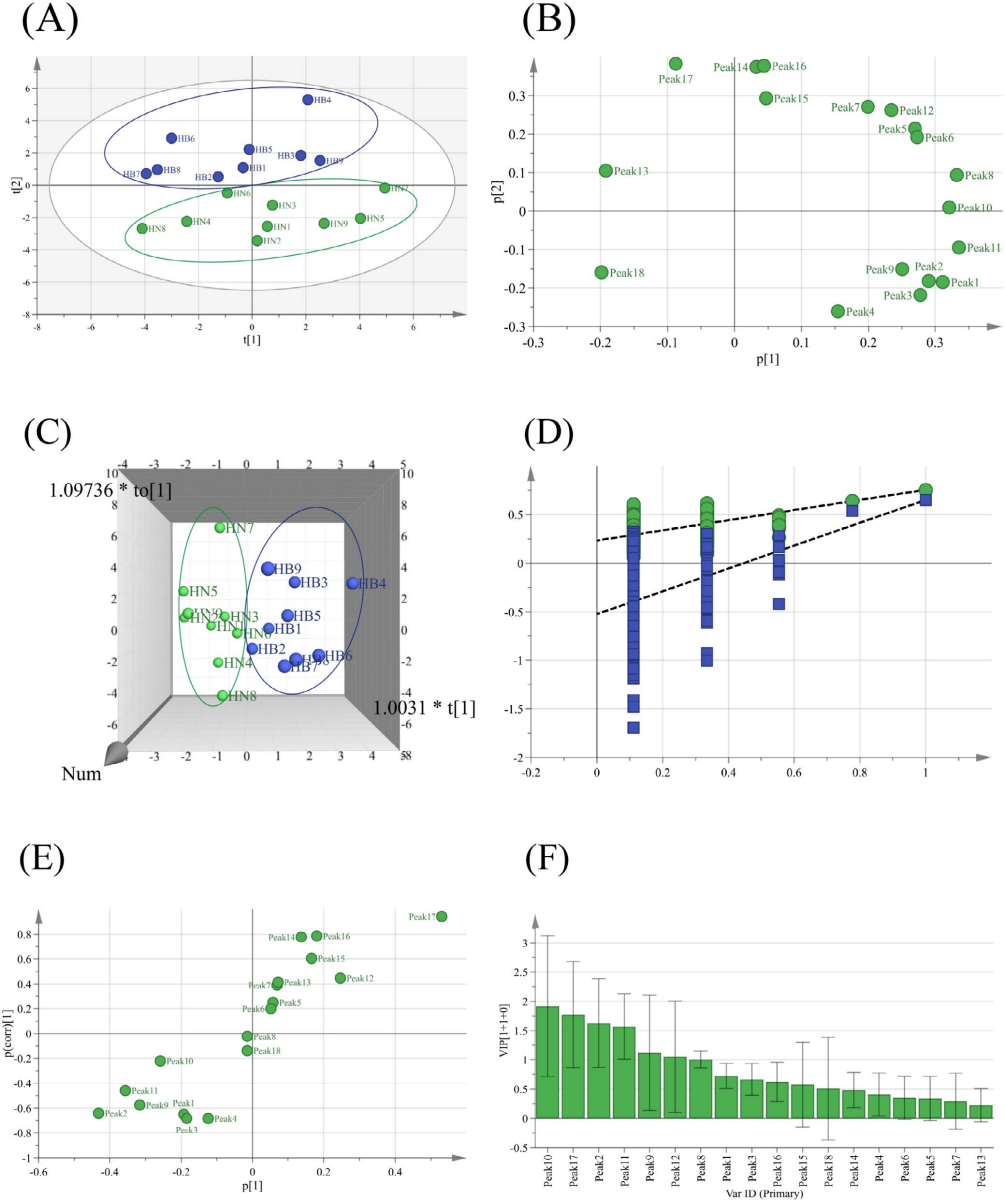
Unsupervised and supervised multivariate data analyses of *Artemisiae argyi* Folium: (A) PCA score plot, (B) loading plot, (C) score 3D plot; (D) permutation test plot, (E) S‐plot, and (F) VIP plot.

Figure 6A shows that the PCA scores of all batches of AAF samples were within the 95% confidence interval, with no outliers detected. The cumulative variance explained by the four principal components (*R* ^2^X) was 0.865, indicating high goodness of fit and reliability. The model effectively represented the information of common peaks in the HPLC‐UV fingerprints. Our results indicate that AAF samples from Henan and Hubei clustered into separate groups, highlighting significant quality variations among AAF from different production areas. Figure 6B demonstrates relatively high loadings for peaks 10 (isochlorogenic acid A), 8 (isochlorogenic acid B), 17 (eupatilin), 12 (3,4,5‐tricaffeoylquinic acid), 2 (chlorogenic acid), 9 (1,5‐*O*‐dicaffeoylquinic acid), 1 (neochlorogenic acid), and 11 (isochlorogenic acid C), which indicates that the levels of these compounds are key factors contributing to quality variations among AAF from different regions. Specifically, peaks 17 (eupatilin) and 12 (3,4,5‐tricaffeoylquinic acid) influenced the clustering of AAF samples from Hubei, with these samples containing significantly higher levels of eupatilin and 3,4,5‐tricaffeoylquinic acid compared with those from Henan. Moreover, peaks 10 (isochlorogenic acid A), 8 (isochlorogenic acid B), 2 (chlorogenic acid), 9 (1,5‐*O*‐dicaffeoylquinic acid), 1 (neochlorogenic acid), and 11 (isochlorogenic acid C) affected the clustering of AAF samples from Henan, which indicates that these samples were rich in these compounds while having relatively low levels of eupatilin and 3,4,5‐tricaffeoylquinic acid.

Supervised orthogonal partial least squares discriminant analysis (OPLS‐DA) was employed for further analysis of the 18 AAF sample batches to identify compounds that significantly influenced the classification of AAF from different production areas and to uncover potential markers contributing to quality variations. The model showed an *R* ^2^X of 0.755 (cumulative explanatory rate of the model in the X‐axis direction), an *R* ^2^Y of 0.950 (cumulative explanatory rate of the model in the Y‐axis direction), and a *Q* ^2^ of 0.651 (predictive power), indicating that the model was stable and effective in prediction (Figure 6C). The 200‐response sorting tests showed that all *R* ^2^ and *Q* ^2^ values obtained through cross‐validation were lower than the original values. The intercept of *R* ^2^ with the Y‐axis was 0.22 (< 0.4) and the intercept of *Q* ^2^ with the Y‐axis was −0.566 (Figure 6D). Therefore, the model was stable and effective without overfitting and could be used for the selection of markers for AAF quality variation. The S‐plot generated from the OPLS‐DA model was used to pinpoint variables that significantly contributed to the variation. Variables situated at both ends of the S‐plot had greater contributions than those in the middle of the plot (Su et al. 2024). The S‐plot indicated that peaks 17 (eupatilin) and 2 (chlorogenic acid) were potential factors influencing the quality variations in AAF samples from the two regions (Figure 6E), which is consistent with the results of unsupervised PCA. Variable importance in projection (VIP) is a critical indicator in the OPLS‐DA model for marker identification, with a high VIP value indicating a significant contribution to the explanatory variables and a strong association with their variations (Ling et al. 2022; Ren et al. 2022).

Selection with VIP > 1 and *p* < 0.05 showed that peaks 10 (isochlorogenic acid A), 17 (eupatilin), 2 (chlorogenic acid), 11 (isochlorogenic acid C), 9 (1,5‐*O*‐dicaffeoylquinic acid), 12 (3,4,5‐tricaffeoylquinic acid), and 8 (isochlorogenic acid B) contributed significantly to the variations in the quality of AAF samples from the two production areas and can be used as effective markers (Figure 6F). This is consistent with the loadings of variables in PCA. Considering these seven markers, we investigated the factors contributing to AAF quality variations. Moreover, quality control was performed to ensure the consistent quality of AAF as a raw material for functional foods.

### 3.3 Establishment and Validation of QAMS for Characteristic Phenylpropanoids and Flavonoids in AAF

QAMS is a simple and cost‐effective quantification method that requires only one internal standard to achieve the simultaneous analysis of multiple active compounds in Chinese herbal medicines or medicinal and edible materials (Duan et al. 2022; Zhang et al. 2021). In this study, a QAMS method was established for measuring six phenylpropanoids and two flavonoids with high abundance in AAF. The compounds included neochlorogenic acid (peak 1), chlorogenic acid (peak 2), cryptochlorogenic acid (peak 3), caffeic acid (peak 4), isochlorogenic acid B (peak 8), isochlorogenic acid C (peak 11), jaceosidin (peak 15), and eupatilin (peak 17). Isochlorogenic acid C, with moderate retention time, optimal peak shape, and low cost, was selected as the internal standard. The standard curves for the six phenylpropanoids and two flavonoids exhibited excellent linear correlations (Table S2). The percentage recovery from the spike‐and‐recovery test varied in the ranges of 95.30%–103.68%, 98.93%–104.46%, 93.35%–100.18%, 94.28%–100.04%, 94.04%–102.41%, 93.76%–98.75%, 94.19%–102.29%, and 95.40%–102.49%. The relative standard deviation (RSD) values were all < 3%, meeting the recovery and precision requirements set by the Association of Official Analytical Chemists. Method validation and robustness assessment were conducted, and the results complied with the criteria outlined in the Chinese Pharmacopeia Chapter 9101 Guidelines for Analytical Method Validation (Jiao et al. 2020). These findings underscored the high specificity, accuracy, and robustness of the established method.

The relative correction factors for neochlorogenic acid (*f*_Neo/Iso C_), chlorogenic acid (*f*_Chl/Iso C_), cryptochlorogenic acid (*f*_Cry/Iso C_), caffeic acid (*f*_Caf/Iso C_), isochlorogenic acid B (*f*_Iso B/Iso C_), jaceosidin (*f*_Jac/Iso C_), and eupatilin (*f*_Eup/Iso C_) were 1.222, 1.178, 1.272, 0.667, 1.006, 1.165, and 1.053, respectively (Table 1). The RSD values were all < 3%. These results indicated that variations in chromatographic column batch, column oven temperature, and flow rate did not significantly affect the correction factors. Therefore, the method demonstrated high robustness under varying conditions. RRT was employed for the peak identification of these compounds. RRT_Neo/Iso C_, RRT_Chl/Iso C_, RRT_Cry/Iso C_, RRT_Caf/Iso C_, RRT_Iso B/Iso C_, RRT_Jac/Iso C_, and RRT_Eup/Iso C_ were 0.219, 0.348, 0.397, 0.454, 0.891, 1.390, and 1.554, respectively (Table 2). Their RSD values were also < 3%. Hence, these compounds exhibited stable RRT values, confirming that the method maintains high system robustness suitable for peak identification.

**TABLE 1:** Relative correction factors of peaks in quantitative analysis of multi‐components by single marker method.

Factor	Effect levels	Relative correction factor, *f*						
		*f*_Neo/Iso C_	*f*_Chl/Iso C_	*f*_Cry/Iso C_	*f*_Caf/Iso C_	*f*_Iso B/Iso C_	*f*_Jac/Iso C_	*f*_Eup/Iso C_
Multi‐point concentration correction	Linear‐1	1.220	1.117	1.360	0.709	0.975	1.078	0.947
	Linear‐2	1.221	1.177	1.310	0.657	1.004	1.188	1.040
	Linear‐3	1.215	1.191	1.269	0.658	1.011	1.166	1.057
	Linear‐4	1.218	1.187	1.269	0.666	1.013	1.182	1.058
	Linear‐5	1.205	1.196	1.275	0.673	1.012	1.164	1.062
	Linear‐6	1.203	1.184	1.260	0.670	1.014	1.153	1.062
	Linear‐7	1.203	1.176	1.259	0.669	1.013	1.149	1.061
Waters CORTECS C_18_	batch‐1	1.236	1.187	1.277	0.672	1.010	1.171	1.064
	batch‐2	1.223	1.176	1.244	0.670	1.004	1.155	1.060
	batch‐3	1.234	1.183	1.276	0.665	1.008	1.197	1.067
HPLC Instrument types	Waters H‐Class	1.283	1.184	1.270	0.659	1.004	1.162	1.061
	SHIMADZU LC‐40D xs	1.218	1.187	1.269	0.666	1.013	1.182	1.058
	Thermo Vanquish	1.272	1.183	1.267	0.671	0.997	1.155	1.058
Column temperature	28°C	1.182	1.175	1.235	0.667	1.002	1.126	1.033
	30°C	1.218	1.187	1.269	0.666	1.013	1.182	1.058
	32°C	1.215	1.174	1.271	0.664	1.013	1.176	1.068
Flow rate	0.38 mL/min	1.223	1.176	1.244	0.670	1.004	1.155	1.060
	0.40 mL/min	1.209	1.168	1.273	0.649	1.004	1.205	1.055
	0.42 mL/min	1.216	1.181	1.276	0.649	1.006	1.181	1.068
Average value		1.222	1.178	1.272	0.667	1.006	1.165	1.053
RSD/%		1.885	1.357	2.019	1.819	0.881	2.356	2.491

*Note:* Neo, Chl, Cry, Caf, Iso B, Iso C, Jac, and Eup stand for neochlorogenic acid, chlorogenic acid, cryptochlorogenic acid, caffeic acid, isochlorogenic acid B, isochlorogenic acid C, jaceosidin, and eupatilin, respectively.

**TABLE 2:** Relative retention time of peaks in quantitative analysis of multi‐components by single marker method.

Factor	Effect levels	Relative retention time/RRT						
		RRT_Neo/Iso C_	RRT_Chl/Iso C_	RRT_Cry/Iso C_	RRT_Caf/Iso C_	RRT_Iso B/Iso C_	RRT_Jac/Iso C_	RRT_Eup/Iso C_
Multi‐point concentration correction	Linear‐1	0.220	0.350	0.399	0.454	0.890	1.388	1.551
	Linear‐2	0.220	0.350	0.399	0.455	0.890	1.388	1.551
	Linear‐3	0.220	0.350	0.398	0.455	0.890	1.390	1.553
	Linear‐4	0.220	0.349	0.398	0.454	0.890	1.389	1.552
	Linear‐5	0.220	0.349	0.398	0.454	0.890	1.389	1.553
	Linear‐6	0.220	0.350	0.399	0.456	0.890	1.388	1.551
	Linear‐7	0.220	0.349	0.398	0.455	0.890	1.390	1.553
Waters CORTECS C_18_	Batch‐1	0.226	0.356	0.404	0.462	0.890	1.388	1.549
	Batch‐2	0.220	0.349	0.397	0.454	0.891	1.393	1.557
	Batch‐3	0.213	0.339	0.386	0.442	0.892	1.402	1.569
HPLC Instrument types	Waters H‐Class	0.219	0.347	0.394	0.452	0.891	1.395	1.559
	SHIMADZU LC‐40D xs	0.220	0.349	0.398	0.454	0.890	1.389	1.552
	Thermo Vanquish	0.219	0.347	0.395	0.453	0.891	1.393	1.557
Column temperature	28°C	0.216	0.352	0.405	0.462	0.893	1.360	1.513
	30°C	0.220	0.349	0.398	0.454	0.890	1.389	1.552
	32°C	0.216	0.348	0.398	0.458	0.896	1.387	1.548
Flow rate	0.38 mL/min	0.220	0.349	0.397	0.454	0.891	1.393	1.557
	0.40 mL/min	0.216	0.342	0.388	0.445	0.893	1.404	1.571
	0.42 mL/min	0.216	0.342	0.389	0.446	0.893	1.404	1.572
Average value		0.219	0.348	0.397	0.454	0.891	1.390	1.554
RSD/%		1.258	1.061	1.144	1.053	0.172	0.643	0.762

*Note:* Neo, Chl, Cry, Caf, Iso B, Iso C, Jac, and Eup stand for neochlorogenic acid, chlorogenic acid, cryptochlorogenic acid, caffeic acid, isochlorogenic acid B, isochlorogenic acid C, jaceosidin, and eupatilin, respectively.

In this study, a novel QAMS method was developed to simultaneously measure six phenylpropanoids and two flavonoids in AAF. The measurement results obtained via QAMS showed no significant differences compared with those obtained via ESM, with RD values < 1% (Table 3). These findings indicate that the QAMS method is accurate and reliable and a suitable alternative to the ESM method for the simultaneous quantification of multiple active compounds in AAF. Moreover, the eupatilin content in AAF from Hubei (1.997 mg/g) was higher than that from Henan (1.053 mg/g), consistent with the chemometric analysis. Therefore, close monitoring and control of eupatilin content during large‐scale production are recommended. This study represents the inaugural application of the QAMS method for the simultaneous quantification of multiple compounds in AAF. The *f* and RRT values successfully passed system validation and were verified using standard substances, confirming their high stability under consistent analytical conditions devoid of background interference. Thus, this method is well‐suited for the quantitative analysis of multiple compounds in AAF raw materials and products and thus eliminates the necessity for repetitive investigations and validations.

**TABLE 3:** Quantification of eight compounds in eighteen batches of *Artemisiae argyi* Folium via quantitative analysis of multi‐components through the single marker method and the external standard method (mg/g).

Batch	Isochlorogenic acid C	Neochlorogenic acid			Chlorogenic acid			Cryptochlorogenic acid			Caffeic acid			Isochlorogenic acid B			Jaceosidin			Eupatilin			Total content		
		ESM	QAMS	RD (%)	ESM	QAMS	RD (%)	ESM	QAMS	RD (%)	ESM	QAMS	RD (%)	ESM	QAMS	RD (%)	ESM	QAMS	RD (%)	ESM	QAMS	RD (%)	ESM	QAMS	RD (%)
HN1	3.567	0.556	0.559	0.270	2.000	1.993	0.158	0.671	0.674	0.223	0.131	0.132	0.699	1.997	1.984	0.307	0.592	0.598	0.448	1.029	1.019	0.503	6.975	6.960	0.112
HN2	3.843	0.598	0.601	0.262	3.250	3.240	0.159	0.570	0.572	0.217	0.108	0.109	0.708	1.596	1.587	0.306	0.464	0.468	0.448	0.988	0.978	0.503	7.574	7.556	0.123
HN3	3.715	0.529	0.532	0.269	2.332	2.325	0.159	0.574	0.577	0.222	0.142	0.144	0.717	1.750	1.739	0.307	0.686	0.692	0.446	1.343	1.330	0.505	7.357	7.339	0.123
HN4	2.283	0.310	0.312	0.259	1.498	1.493	0.160	0.357	0.358	0.219	0.095	0.096	0.721	0.864	0.859	0.308	0.479	0.483	0.443	1.085	1.074	0.502	4.688	4.676	0.130
HN5	4.237	0.616	0.620	0.264	2.482	2.474	0.159	0.577	0.579	0.223	0.116	0.118	0.704	2.492	2.477	0.307	0.549	0.554	0.445	0.601	0.595	0.500	7.433	7.416	0.113
HN6	2.764	0.367	0.369	0.266	1.623	1.618	0.157	0.354	0.356	0.213	0.049	0.049	0.693	1.614	1.604	0.304	0.525	0.530	0.445	1.189	1.178	0.502	5.721	5.703	0.157
HN7	4.977	0.760	0.764	0.269	3.786	3.774	0.159	0.576	0.578	0.223	0.089	0.090	0.693	3.621	3.599	0.306	0.610	0.615	0.441	1.476	1.461	0.503	10.918	10.882	0.163
HN8	1.571	0.207	0.208	0.278	0.603	0.601	0.158	0.278	0.279	0.228	0.134	0.136	0.725	0.668	0.664	0.306	0.441	0.444	0.449	0.917	0.908	0.502	3.247	3.240	0.105
HN9	3.974	0.566	0.569	0.265	2.237	2.230	0.157	0.594	0.597	0.225	0.127	0.129	0.698	1.823	1.812	0.305	0.462	0.466	0.442	0.850	0.841	0.503	6.658	6.643	0.113
HB1	2.577	0.374	0.376	0.273	1.264	1.260	0.158	0.337	0.339	0.221	0.043	0.044	0.754	1.571	1.561	0.305	0.446	0.450	0.445	1.566	1.550	0.503	5.601	5.580	0.188
HB2	2.170	0.260	0.261	0.269	1.571	1.566	0.156	0.281	0.282	0.225	0.070	0.071	0.736	1.253	1.245	0.306	0.527	0.531	0.442	1.419	1.405	0.504	5.380	5.361	0.171
HB3	2.943	0.342	0.343	0.257	1.568	1.563	0.160	0.331	0.333	0.216	0.066	0.067	0.694	2.449	2.434	0.306	0.569	0.575	0.451	1.835	1.816	0.504	7.159	7.130	0.204
HB4	3.024	0.293	0.294	0.269	1.165	1.161	0.159	0.335	0.337	0.220	0.086	0.087	0.684	2.598	2.582	0.308	0.883	0.891	0.443	2.379	2.355	0.503	7.738	7.707	0.203
HB5	3.152	0.304	0.306	0.268	1.185	1.181	0.161	0.355	0.357	0.218	0.084	0.085	0.717	1.688	1.678	0.306	0.526	0.531	0.447	1.873	1.854	0.502	6.016	5.992	0.197
HB6	1.453	0.213	0.214	0.255	0.693	0.691	0.159	0.183	0.184	0.214	0.038	0.038	0.779	0.998	0.991	0.307	0.943	0.951	0.446	2.557	2.531	0.502	5.624	5.601	0.204
HB7	2.033	0.171	0.172	0.276	0.607	0.605	0.157	0.221	0.221	0.225	0.032	0.032	0.640	1.202	1.195	0.307	0.576	0.581	0.446	2.155	2.133	0.502	4.962	4.939	0.235
HB8	2.113	0.181	0.182	0.257	0.642	0.640	0.164	0.243	0.244	0.213	0.032	0.033	0.707	1.372	1.364	0.309	0.592	0.597	0.449	2.168	2.147	0.504	5.231	5.206	0.235
HB9	4.758	0.583	0.586	0.269	2.377	2.370	0.158	0.607	0.610	0.217	0.085	0.086	0.681	2.780	2.763	0.306	0.796	0.803	0.450	2.020	2.000	0.501	9.248	9.217	0.165

## 4 Conclusions

In this study, a novel method based on HPLC‐Q‐Exactive Orbitrap‐HRMS was developed for the rapid identification of chemical compounds in AAF. This method was used to elucidate pharmacodynamically active compounds in AAF, predominantly flavonoids and phenylpropanoids. We highlighted the characteristic fragmentation pathways of these compounds, laying a foundation for the structural identification of analogous compounds in other medicinal and edible materials. Furthermore, we generated HPLC‐UV fingerprints of AAF from various regions in China and identified potential markers for quality variations using a chemometric approach. To enhance the quality control of AAF, a QAMS method was developed for the simultaneous quantification of six phenylpropanoids and two flavonoids, compounds abundant in AAF. This method is characterized by accuracy, reliability, and efficiency, significantly reducing analysis time and research costs while improving analytical efficiency. This study provides a scientific and efficient analytical method and a scientific basis for the standardized production, component analysis, and quality control of AAF, an important traditional medicinal and edible material. These findings also serve as a valuable reference for quality control in similar medicinal and edible materials.

## Author Contributions

**Feifei Xie:** methodology, data curation, formal analysis, investigation, and writing – original draft. **Cong Ye:** methodology, data curation, formal analysis, investigation, and writing – original draft. **Haibao Qiu**, **Xiaoying Lu** and **Liyuan Qu:** formal analysis, investigation, and writing – review and editing. **Zhizhou Ling, Zhenyu Li** and **Dongmei Sun:** methodology, data curation. **Lin Zhou**, **Minyou He** and **Wenhui Luo:** conceptualization, data curation, supervision, and validation.

## Conflicts of Interest

The authors declare no conflicts of interest.

## Supporting information


**Table S1:** Compounds identified in *Artemisiae argyi* Folium based on HPLC‐Q‐Exactive Orbitrap‐HRMS.
**Table S2:** Regression equation, correlation coefficient and linear range of eight compounds.

## Data Availability

The authors have nothing to report.
