# Supplementary material for: Research on Material Basis and Quality Control of Artemisiae argyi Folium, a Traditional Medicinal and Edible Material
Source: Food Sci Nutr. 2025 Sep 1;13(9):e70856. doi: 10.1002/fsn3.70856 (PMC12400158; doi:10.1002/fsn3.70856)
Supplement: Supplementary file 1 — Table S1: Compounds identified in Artemisiae argyi Folium based on HPLC‐Q‐Exactive Orbitrap‐HRMS. Table S2: Regression equation, correlation coefficient and linear range of eight compounds. [file FSN3-13-e70856-s001.docx]

**Table S1** Compounds identified in Artemisiae argyi Folium based on HPLC-Q-Exactive Orbitrap-HRMS.

| **No.** | tR (min) | **Type of fragment ions** | Quasi-molecular ion formula | Theoretical mass (m/z) | Experimental mass (m/z) | Error (×10−6) | Main characteristic fragment ion (m/z) | **Identification** | **References** |
| --- | --- | --- | --- | --- | --- | --- | --- | --- | --- |
| 1 | / | [M+H]+ | / | / | / | / | / | Quinic acid | / |
|  | 3.10 | [M-H]− | C7H11O6 | 191.05556 | 191.05531 | -1.31 | 191.05531, 173.04451, 93.03324, 85.02808 |  |  |
| 2 | 5.97 | [M+H]+ | C9H12NO2 | 166.08681 | 166.08649 | -1.93 | 120.08105, 103.05470 | L-Phenylalanine | Wang et al., 2020 |
|  | 6.02 | [M-H]− | C9H10NO2 | 164.07115 | 164.07118 | 0.18 | 147.04424, 96.96832, 78.95775, 72.00775 |  |  |
| 3 | 9.99 | [M+H]+ | C16H19O9 | 355.10291 | 355.10239 | -1.46 | 163.03896, 145.02824, 135.04385 | Neochlorogenic acid | Standard |
|  | 9.92 | [M-H]− | C16H17O9 | 353.08725 | 353.08835 | 3.03 | 191.05551, 179.03426, 173.04533, 135.04398 |  |  |
| 4 | 10.58 | [M+H]+ | C11H13N2 | 205.09771 | 205.0975 | -1.02 | 188.07101, 159.09209, 146.0603, 118.06554 | L-Tryptophan | / |
|  | 10.68 | [M-H]− | C11H11N2 | 203.08205 | 203.08174 | -1.53 | 203.08174, 142.06506.116.04931, 74.02337 |  |  |
| 5 | 16.21 | [M+H]+ | C16H19O9 | 355.10291 | 355.10229 | -1.75 | 163.03902, 145.02852, 135.04411, 117.03419 | Chlorogenic acid | Standard |
|  | 16.23 | [M-H]− | C16H17O9 | 353.08725 | 353.08798 | 2.07 | 191.05556, 179.03447, 135.21600 |  |  |
| 6 | 18.10 | [M+H]+ | C16H19O9 | 355.10291 | 355.10251 | -1.13 | 163.03909, 145.02853, 135.04440, 117.03366 | Cryptochlorogenic acid | Standard |
|  | 18.03 | [M-H]− | C16H17O9 | 353.08725 | 353.08813 | 2.49 | 191.05550, 179.03423, 173.04474, 135.04414 |  |  |
| 7 | / | [M+H]+ | / | / | / | / | / | Caffeic acid | Standard |
|  | 19.72 | [M-H]− | C9H7O4 | 179.03443 | 179.03423 | -1.12 | 135.04395, 107.04884, 78.95763 |  |  |
| 8 | 23.44 | [M+H]+ | C27H31O15 | 595.1663 | 595.16656 | 0.44 | 421.08920, 403.08414, 379.08179, 337.07111, 325.07089, 295.06049 | Vicenin 2 | Standard |
|  | 23.45 | [M-H]− | C27H29O15 | 593.15064 | 593.15222 | 2.66 | / |  |  |
| 9 | 26.27 | [M+H]+ | C26H29O14 | 565.15574 | 565.15576 | 0.04 | 511.90494, 433.09396, 379.08160, 325.07062 | Schaftoside | Standard |
|  | 26.31 | [M-H]− | C26H27O14 | 563.14008 | 563.14136 | 2.27 | 473.10779, 443.09933, 383.07761, 353.06717, 332.80417, 325.07254, 297.07742 |  |  |
| 10 | 27.59 | [M+H]+ | C26H29O14 | 565.15574 | 565.15576 | 0.04 | 379.08163, 349.07068, 337.07098, 325.07004, 307.06015, 295.06052, | Neoshaftoside | Standard |
|  | 27.66 | [M-H]− | C26H27O14 | 563.14008 | 563.14154 | 2.59 | 383.07770, 353.06702, 325.07254, 297.07703 |  |  |
| 11 | 28.11 | [M+H]+ | C26H29O14 | 565.15574 | 565.15594 | 0.35 | 379.08109, 337.07086, 325.07089, 295.06046 | Vicenin 3 | Standard |
|  | 28.12 | [M-H]− | C26H27O14 | 563.14008 | 563.14142 | 2.38 | 383.07733, 365.06674, 353.06671, 332.80350, 325.06982 |  |  |
| 12 | 30.21 | [M+H]+ | C27H31O16 | 611.16121 | 611.16125 | 0.07 | 303.05005, 274.14041, 137.02341, 97.02892, 85.02899 | Rutin | Standard |
|  | 30.20 | [M-H]− | C27H29O16 | 609.14555 | 609.14697 | 2.33 | 301.03363, 300.02774, 178.99791, 151.00278 |  |  |
| 13 | 30.33 | [M+H]+ | C21H21O10 | 433.11348 | 433.11334 | -0.32 | 379.08142, 337.07123, 313.07086, 283.06033 | Vitexin | Han et al., 2017 |
|  | 30.23 | [M-H]− | C21H19O10 | 431.09782 | 431.09877 | 2.2 | 376.86981, 341.06744, 311.05655, 283.06119, 269.04532 |  |  |
| 14 | 30.50 | [M+H]+ | C27H31O15 | 595.1663 | 595.16663 | 0.55 | 287.05511 | Luteolin 7-O-rutinoside | / |
|  | 30.54 | [M-H]− | C27H29O15 | 593.15064 | 593.1521 | 2.46 | 285.04056 |  |  |
| 15 | 31.43 | [M+H]+ | C15H11O7 | 303.05048 | 303.05011 | -1.22 | 285.03958, 257.04422, 229.04971, 219.02927, 183.02924, 153.01848 | Quercetin | / |
|  | / | [M-H]− | / | / | / | / | / |  |  |
| 16 | 31.46 | [M+H]+ | C21H21O12 | 465.10331 | 465.10333 | 0.04 | 303.05002, 85.02890 | Isoquercetin | Standard |
|  | 31.47 | [M-H]− | C21H19O12 | 463.08765 | 463.08887 | 2.63 | 301.03412, 300.02783, 178.99759, 151.00258 |  |  |
| 17 | 31.66 | [M+H]+ | C21H21O11 | 449.10839 | 449.10815 | -0.53 | 287.05515, 270.89209 | Cynaroside | Standard |
|  | 31.59 | [M-H]− | C21H19O11 | 447.09273 | 447.09433 | 3.58 | 285.04034, 250.86649 |  |  |
| 18 | 34.38 | [M+H]+ | C25H25O12 | 517.13461 | 517.13452 | -0.17 | 163.03905, 145.02846, 135.04404, 117.03371 | Isochlorogenic acid B | Standard |
|  | 34.33 | [M-H]− | C25H23O12 | 515.11895 | 515.1203 | 2.04 | 353.08841, 335.07819, 191.05560, 179.03424, 173.04474, 135.04384 |  |  |
| 19 | 35.52 | [M+H]+ | C25H25O12 | 517.13461 | 517.13452 | -0.17 | 163.03908, 145.02859, 135.04416, 117.03380 | 1,5-O-Dicaffeoylquinic acid | Standard |
|  | 35.59 | [M-H]− | C25H23O12 | 515.11895 | 515.12 | 2.04 | 353.08856, 335.07825, 191.05557, 179.03439 |  |  |
| 20 | 36.03 | [M+H]+ | C25H25O12 | 517.13461 | 517.13422 | -0.75 | 163.03909, 145.02856, 135.04420, 117.03370 | Isochlorogenic acid A | Standard |
|  | 35.99 | [M-H]− | C25H23O12 | 515.11895 | 515.11981 | 1.67 | 353.08847, 191.05556, 179.03424, 173.04457, 135.04422 |  |  |
| 21 | / | [M+H]+ | / | / | / | / | / | Azelaic acid | Han et al., 2017 |
|  | 37.54 | [M-H]− | C9H15O4 | 187.09703 | 187.09677 | -1.39 | 130.49634, 125.09595, 97.06454, 78.95764 |  |  |
| 22 | 37.84 | [M+H]+ | C21H19O11 | 447.09274 | 447.09247 | -0.6 | 271.06018 | Apigenin-7-glucuronide | / |
|  | 37.80 | [M-H]− | C21H17O11 | 445.07708 | 445.07806 | 2.2 | 342.81253, 310.82181, 269.04578, 248.86565, 230.85489 |  |  |
| 23 | 38.81 | [M+H]+ | C25H25O12 | 517.13461 | 517.13434 | -0.52 | 163.03908, 145.02850, 135.04431, 117.03382 | Isochlorogenic acid C | Standard |
|  | 38.77 | [M-H]− | C25H23O12 | 515.11895 | 515.11987 | 1.79 | 353.08838, 191.05533, 179.03419, 173.04477, 135.04436 |  |  |
| 24 | / | [M+H]+ | / | / | / | / | / | Hydroxybenzoic acid | / |
|  | 38.97 | [M-H]− | C7H5O3 | 137.02386 | 137.02325 | -4.45 | 93.03321 |  |  |
| 25 | 43.99 | [M+H]+ | C28H33O14 | 593.18704 | 593.18663 | -0.69 | 285.07596, 85.02910 | Linarin | / |
|  | / | [M-H]− | / | / | / | / | / |  |  |
| 26 | 44.16 | [M+H]+ | C16H13O5 | 285.0763 | 285.07593 | -1.3 | 270.05194 | Acacetin | / |
|  | / | [M-H]− | / | / | / | / | / |  |  |
| 27 | 45.24 | [M+H]+ | C15H13O6 | 289.07122 | 289.07086 | -1.25 | 179.03421, 171.02904, 163.03908, 153.01837, 145.02840 | Eriodictyol | Cui et al., 2021 |
|  | 45.28 | [M-H]− | C15H11O6 | 287.05556 | 287.05646 | 3.14 | 188.86194, 151.00270, 135.04391, 107.01247 |  |  |
| 28 | 45.62 | [M+H]+ | C15H11O6 | 287.05557 | 287.05511 | -1.6 | 153.01894 | Luteolin | Standard |
|  | 45.72 | [M-H]− | C15H9O6 | 285.03991 | 285.04065 | 2.6 | 151.00275, 149.02338, 107.01257 |  |  |
| 29 | 45.82 | [M+H]+ | C16H13O7 | 317.06613 | 317.06583 | -0.95 | 302.04227 | Nepetin | Cui et al., 2021 |
|  | 45.75 | [M-H]− | C16H11O7 | 315.05047 | 315.05142 | 3.02 | 198.91728, 136.98692, 96.96824, 78.95765 |  |  |
| 30 | 46.20 | [M+H]+ | C34H31O15 | 679.1663 | 679.16644 | 0.21 | 163.03909, 145.02856, 135.04399 | 3,4,5-Tricaffeoylquinic acid | / |
|  | 46.27 | [M-H]− | C34H29O15 | 677.15064 | 677.15515 | 2.33 | 515.12018, 353.08829, 335.07828, 191.05644, 179.03407, 173.04524 |  |  |
| 31 | 48.23 | [M+H]+ | C16H13O7 | 317.06613 | 317.06583 | -0.95 | 302.04233, 275.05576, 225.03979, 111.04417 | Capillarisin | Cui et al., 2021 |
|  | / | [M-H]− | / | / | / | / | / |  |  |
| 32 | 50.06 | [M+H]+ | C15H11O5 | 271.06065 | 271.06024 | -1.51 | 153.0184 | Apigenin | Standard |
|  | 49.99 | [M-H]− | C15H9O5 | 269.04499 | 269.04572 | 2.71 | 151.00244, 149.02330, 107.01252 |  |  |
| 33 | / | [M+H]+ | / | / | / | / | / | Naringenin | Standard |
|  | 50.16 | [M-H]− | C15H11O5 | 271.06064 | 271.06183 | 4.39 | 177.01842, 151.00270, 119.04907, 107.01257, 93.03330 |  |  |
| 34 | 50.37 | [M+H]+ | C16H13O6 | 301.07122 | 301.07071 | -1.69 | 286.04724, 254.05739 | Hispidulin | Cui et al., 2021 |
|  | 50.44 | [M-H]− | C16H11O6 | 299.05556 | 299.05637 | 2.71 | 284.03290, 136.98686 |  |  |
| 35 | 50.77 | [M+H]+ | C16H13O6 | 301.07122 | 301.07071 | -1.69 | 286.04742 | Diosmetin | Standard |
|  | 50.76 | [M-H]− | C16H11O6 | 299.05556 | 299.05646 | 3.01 | 284.03284 |  |  |
| 36 | 51.12 | [M+H]+ | C17H15O7 | 331.08178 | 331.08124 | -1.63 | 316.05792, 301.03394 | Jaceosidin | Standard |
|  | 51.05 | [M-H]− | C17H13O7 | 329.06612 | 329.06708 | 2.92 | 314.04355, 271.02502, 299.01993 |  |  |
| 37 | 52.00 | [M+H]+ | C18H17O8 | 361.09235 | 361.09201 | -0.94 | 346.06839, 328.05817, 361.09201, 300.06284 | Irigenin | Standard |
|  | / | [M-H]− | / | / | / | / | / |  |  |
| 38 | 52.90 | [M+H]+ | C18H17O8 | 361.09235 | 361.09201 | -0.94 | 361.09192, 346.06815, 328.05823, 311.05545, 300.06216 | 5,7,3'-Trihydroxy-6,4',5'-trimethoxyflavone | Cui et al., 2021 |
|  | 52.97 | [M-H]− | C18H15O8 | 359.07669 | 359.07761 | 2.56 | 344.05316, 329.03049, 314.00763, 286.01212 |  |  |
| 39 | 53.07 | [M+H]+ | C18H17O8 | 361.09235 | 361.09174 | -1.69 | 361.09192, 346.06824, 300.06299, 328.05768 | Centaureidin | Standard |
|  | 53.17 | [M-H]− | C18H15O8 | 359.07669 | 359.07758 | 2.48 | 343.04675, 329.03061, 314.00711, 301.03488 |  |  |
| 40 | 55.97 | [M+H]+ | C17H15O6 | 315.08687 | 315.08655 | -1.02 | 299.05536, 282.05231, 271.06000, 254.05733 | Cirsimaritin | Hossain et al., 2010 |
|  | 55.85 | [M-H]− | C17H13O6 | 313.07121 | 313.07184 | 2.01 | 298.04797, 283.02524, 269.04684, 255.02946 |  |  |
| 41 | 56.62 | [M+H]+ | C19H19O8 | 345.09743 | 345.09723 | -0.58 | 330.07358, 315.04910, 287.05338 | Eupatilin | Standard |
|  | 56.64 | [M-H]− | C19H17O8 | 343.08177 | 343.08255 | 2.27 | 328.05917, 313.03577, 298.01181, 285.03986 |  |  |
| 42 | 57.55 | [M+H]+ | C20H20O8 | 389.12365 | 389.1234 | -0.64 | 374.09946, 359.07666, 356.08945, 331.08139 | Artemetin | Standard |
|  | / | [M-H]− | / | / | / | / | / |  |  |
| 43 | 58.00 | [M+H]+ | C19H19O8 | 375.108 | 375.10776 | -0.64 | 360.08368, 342.07379, 317.06577, 299.05518, 75.10794 | Chrysosplenetin B | Standard |
|  | 58.07 | [M-H]− | C19H17O8 | 373.09234 | 373.09302 | 1.82 | 358.06906, 343.04617, 328.02277, 300.02820 |  |  |
| 44 | 58.34 | [M+H]+ | C19H19O8 | 375.108 | 375.1076 | -1.07 | 359.07672, 342.07394, 317.06610, 299.05508 | Casticin isomer | Han et al., 2017 |
|  | 58.26 | [M-H]− | C19H17O8 | 373.09234 | 373.09327 | 2.49 | 358.07040, 343.04602, 328.02252, 315.05145, 300.02774, 285.00452 |  |  |
| 45 | 59.54 | [M+H]+ | C17H14O6 | 315.08687 | 315.08649 | -1.21 | 315.08633, 300.06308 | Pectolinarigenin | Standard |
|  | / | [M-H]− | / | / | / | / | / |  |  |

Note: “/”represents below the detection limit.

**Table S2** Regression equation, correlation coefficient and linear range of 8 compounds.

| **Compound** | **Regression equation** | **r** | **Linear range, μg/mL** |
| --- | --- | --- | --- |
| Neochlorogenic acid | *y*=16035x-69.17 | 1 | 0.409-40.925 |
| Chlorogenic acid | *y*=16372x-1320.5 | 1 | 1.540-153.964 |
| Cryptochlorogenic acid | *y*=15330x-407.28 | 1 | 0.400-39.984 |
| Caffeic acid | *y*=28815x+ 275.07 | 1 | 0.211-21.136 |
| Isochlorogenic acid B | *y*=19027x+1198.9 | 1 | 1.174-117.365 |
| Isochlorogenic acid C | *y*=19279x+1957.2 | 1 | 2.445-244.451 |
| Eupatilin | *y*=16786x-662.31 | 1 | 0.425-42.454 |
| Ligupurpuroside B | *y*=18155x+ 1724.3 | 1 | 1.199-119.913 |

**References**

Cui, L., Wang, X., Lu, J., Tian, J., Wang, L., Qu, J., Liu, Z., & Wei, J. (2021). Rapid identification of chemical constituents in *Artemisia argyi* lévi. et vant by UPLC-Q-exactive-MS/MS. *Journal of Food Quality*, *2021*, 5597327. https://doi.org/10.1155/2021/5597327

Han, B., Xin, Z., Ma, S., Liu, W., Zhang, B., Ran, L., Yi, L.& Ren, D. (2017). Comprehensive characterization and identification of antioxidants in *Folium Artemisiae Argyi* using high-resolution tandem mass spectrometry. *Journal of chromatography. B, Analytical technologies in the biomedical and life sciences, 1063*, 84–92. https://doi.org/10.1016/j.jchromb.2017.08.021

Hossain, M. B., Rai, D. K., Brunton, N. P., Martin-Diana, A. B., & Barry-Ryan, C. (2010). Characterization of phenolic composition in Lamiaceae spices by LC-ESI-MS/MS. *Journal of agricultural and food chemistry*, *58*(19)*,* 10576–10581. https://doi.org/10.1021/jf102042g

Wang, X., Zhong, X., Zhou, N., Cai, N., Xu, J., Wang, Q., Li J., Liu, Q., Lin, Q., & Shang, X. (2020). Rapid Characterizaiton of Chemical Constituents of the Tubers of *Gymnadenia conopsea* by UPLC-Orbitrap-MS/MS Analysis. *Molecules (Basel, Switzerland)*, *25*(4), 898. https://doi.org/10.3390/molecules25040898
